# Differential responses to photoperiod in juveniles of two migratory songbird species

**DOI:** 10.1242/jeb.252259

**Published:** 2026-07-14

**Authors:** Clara D'Autilia, Giuseppe Bianco, Susanne Åkesson

**Affiliations:** Department of Biology, Lund University, 22362 Lund, Sweden

**Keywords:** Endogenous migratory programme, Fuelling, Migratory restlessness, Photoperiod, European robin, Garden warbler

## Abstract

Songbirds rely on an endogenous programme encoding spatio-temporal information necessary for naive individuals to perform their first migration. Timing programmes are generated by endogenous clocks and include responses to external cues, with photoperiod being the most important. Photoperiod can advance or delay the circannual cycle and, while it is well established as a trigger for initiating migration, it remains uncertain whether birds continue to integrate photoperiod into their programme during migration. This study investigated whether two passerine species incorporate photoperiod in their endogenous programmes during autumn migration. Photoperiod manipulation experiments were conducted on hatch-year individuals of European robin (*Erithacus rubecula*; short-to-medium distance migrant) and garden warbler (*Sylvia borin*; long-distance migrant), monitoring their migratory activity and fuelling. The two species responded differently to the treatment, possibly due to different migratory distances, though effects of stage-appropriate responses cannot be excluded. European robins were partially sensitive to photoperiodic cues regarding their migratory activity. Their fuelling was, however, unaffected by photic treatment. Garden warblers showed little sensitivity to photoperiod, suggesting perhaps a stronger reliance on other external cues during migration. Garden warblers, furthermore, exhibited an exceptionally high fuelling rate, compared with other species previously studied, probably an adaptation to their time-constrained, long trans-equatorial migration. The results suggest that different components of the migratory phenotype can be controlled by different cues, with species-specific differences in the response. Understanding how migratory timing programmes interact with environmental cues in various migration phases is crucial, especially as anthropogenic processes rapidly alter photic environments, risking threatening migratory birds.

## Teaser

**Summary:** Daylength manipulation has differential effects on the migratory activity and fuelling of two songbird species, the European robin and the garden warbler, indicating species-specific effects of photic cues on the migratory phenotype.

## INTRODUCTION

Avian migration has been studied for centuries (Berthold, 2001), but there are still open questions related to the time and space programmes controlling it. Two key aspects of migration are orientation in space and time keeping, i.e. how birds manage to align their migration, with the related processes and behaviours, to the annual cycle (Helm and Liedvogel, 2024). The mechanisms controlling migration phenotype have a genetic basis and use endogenous clocks and responses to external cues to generate adaptive timing programmes (Kramer, 1957; Åkesson et al., 2017).

Time keeping in migratory birds relies on two endogenous clocks, a circadian and a circannual clock (Aschoff, 1960; Berthold, 1973; Gwinner, 1996). Experiments with captive birds have shown that the circannual rhythm is important for triggering not only the onset of migration but also other processes preceding and following migration, such as pre-migratory fuelling (Berthold et al., 1972; Gwinner, 1986; Kumar et al., 2006). Migratory fuelling is the accumulation of energy reserves, primarily as fat, in preparation for migration (King and Farner, 1963; Alerstam, 1990; Hedenström and Alerstam, 1997). The time ratio between fuelling and flying has been theoretically determined to approach 7:1 (Hedenström and Alerstam, 1997). It has been shown that the diurnal periodicity of migratory restlessness and other related processes is controlled by an endogenous circadian clock (Gwinner, 1975; McWilliams et al., 2022).

Most songbirds migrate at night, transitioning from being diurnally active to expressing nocturnal migratory restlessness (in German ‘Zugunruhe’), a behaviour involving flying, hopping and whirring the wings, when studied in cages (Berthold, 2001). As migratory restlessness is generally synchronous with the migration of free-living birds and is a directed behaviour (Kramer, 1949; Gwinner and Wiltschko, 1978), it has been widely used as a proxy to study migration. The length of the migratory restlessness period has also been shown to be correlated with the distance a species covers during its natural migration (Berthold, 1973, 1988; Gwinner, 1986).

Migratory restlessness, however, is only a window open for migration in the birds' endogenous time programme (Gwinner and Czeschlik, 1978) and the actual phases of migration in the wild, including fuelling, are triggered or inhibited by external factors, such as photoperiod, geomagnetic cues, wind and availability of food and stopover sites (Åkesson and Hedenström, 2000; Fransson et al., 2001; Jenni and Schaub, 2003; Helm et al., 2009; Åkesson et al., 2017; Ilieva et al., 2018).

Among environmental cues, photoperiod, i.e. the annually changing light portion of the day, has the greatest impact on endogenous timing programmes and can both advance and delay the circannual cycle and, thus, migration (Rowan, 1926; Lindström et al., 1994; Helm et al., 2005; Senner et al., 2014; Briedis et al., 2018; Åkesson and Helm, 2020). Nevertheless, not many experiments have recorded how photoperiod affects Zugunruhe and fuelling simultaneously. Åkesson et al. (2021) focused on the effect of day length on fuelling and the onset, level and extent of migratory restlessness in two temperate zone passerine migrants, the dunnock (*Prunella modularis*) and the European robin (*Erithacus rubecula*). The results showed the birds' sensitivity to photoperiod manipulation and their ability to reset their activity patterns according to the new diel cycle. This study highlighted the fact that different parts of the migratory phenotype can be either under strict endogenous control or more flexible to external cues, but also that there may be species-specific differences in reaction norms.

Although there is strong evidence that photoperiod is an important cue for initiating migration, it is still unknown whether birds use photoperiod during their migration, especially when migrating for the first time, integrating it into their endogenous programme. This would be possible, as photoperiod changes predictably with date and latitude, and could consequently provide temporal and positional information (Huffeldt, 2020; Sockman and Hurlbert, 2020).

A fundamental characteristic of timing programmes is that the encoded responses to external cues are specific to the phase of the annual cycle (Åkesson and Helm, 2020). This means all the different stages of migration need to be investigated independently to get a complete understanding of how this complex phenomenon is regulated.

Huffeldt et al. (2024) conducted a photoperiod manipulation experiment on captive hatch-year dunnocks, monitoring their fuelling and activity, and found that photoperiod is incorporated into the endogenous programme during migration, though the effect on dunnocks was different from what Bojarinova and Babushkina (2015) had observed for long-tailed tits (*Aegithalos caudatus caudatus*). Given the scarcity of research, it is still unconfirmed whether the integration of photoperiod into migratory programmes during migration is a ubiquitous trait in birds or not (Huffeldt et al., 2024). Now more than ever, it is important to investigate how migration programmes are affected by the photic environment, as light conditions are rapidly shifting due to anthropogenic processes.

Here, we studied whether two species of songbirds with different migratory distances incorporated photoperiod into their expression of endogenous programmes during migration, by continuously monitoring their activity and fuelling in cages. Photoperiod manipulation experiments were conducted on two nocturnal songbird migrants, the garden warbler (*Sylvia borin*) and the European robin (hereafter robin). The experiments were performed during their autumn migration using the same set-up. The birds, all hatch-year individuals, were divided into a control and an experimental group and subjected to an artificial photoperiod for approximately 2 weeks: the control group experienced the local photoperiod (southern Sweden), while the experimental group was exposed to the photoperiod of southern France, thus being virtually displaced south.

We hypothesised that the photoperiod manipulation would affect activity and fuelling, traits associated with the migratory phenotype (Lindström et al., 1994; Åkesson et al., 2021; Huffeldt et al., 2024). We wanted to study species with different migration distances, as they may react differently to day-length cues, the garden warbler being a long-distance trans-equatorial migrant and the robin migrating short-to-medium distances (Fransson and Hall-Karlsson, 2008). For the garden warbler, southern France and south thereof is a stopover site before the crossing of the Mediterranean Sea and the Sahara Desert, while for the robin, southern France represents the migration goal (Fransson and Hall-Karlsson, 2008).

We predicted that both control and virtually displaced garden warblers would be highly motivated to migrate, i.e. that the migratory restlessness would be retained and comparable in the two groups, as the control birds were still at the beginning of their migration and the displaced birds at a later stopover site. Regarding the fuelling, we had two alternative hypotheses for garden warblers: again, we expected both groups to be highly motivated to fuel, but the experimental group, displaced to southern France, could either (1) show the same fuelling rate as the control group, or (2) show a higher one, as would be expected before an ecological barrier (Fransson et al., 2001). For robins, we predicted that the virtually displaced (experimental) birds would reduce both their migratory restlessness and fuelling, compared with the control birds, if they interpreted the new photic conditions as being spatially displaced to their wintering grounds.

In short, the same manipulation placed each species at a functionally different migratory stage, limiting the possibility to directly compare results from the two species.

## MATERIALS AND METHODS

### Study species

This study involved two passerine species, both nocturnal migrants: the garden warbler, *Sylvia borin* (Boddaert 1783), and the European robin, *Erithacus rubecula* (Linnaeus 1758).

The garden warbler is a long-distance migrant that breeds in large parts of Europe and winters in southern Europe and Sub-Saharan Africa (Gwinner and Wiltschko, 1978; Bairlein, 1987). Ringing data show that the majority of Scandinavian garden warblers pass through southern Sweden between August and the beginning of September and have left Europe by October, spending the winter in western to southern Sub-Saharan Africa; their average migration speed has been estimated by ringing recoveries to be approximately 65 km day^−1^ (Fransson and Hall-Karlsson, 2008). A study by Ottosson et al. (2005) identified the mean date of Scandinavian garden warblers reaching the southern edge of the Sahara as early as the beginning of October, although these estimates could be changing due to climate change.

The European robin is a short-to-medium distance migrant, breeding in Europe and wintering in the Mediterranean region. Scandinavian robins start their autumn migration in September and have a winter distribution covering substantial parts of southern Europe, with most individuals wintering in Italy, southern France and the Iberian Peninsula (Pettersson and Lindholm, 1983; Fransson and Hall-Karlsson, 2008). Recovery data show that the robin has a relatively slow migration speed overall, taking about 36 days to migrate from Ottenby (southern Sweden) to the Iberian Peninsula (Pettersson and Hasselquist, 1985), which is connected to the finding that migratory fuelling in this species is relatively slow (Pettersson, 1983 in Pettersson and Hasselquist, 1985). The migration speed for robins has also been estimated based on ringing recoveries to ca 53.2 km day^−1^ (Fransson and Hall-Karlsson, 2008).

### Experimental birds and experimental facility

Juveniles of both species were captured during their autumn migration near Ystad, southern Sweden (55°25′N, 13°49′E). After capture, the birds were ringed and transported by car to Lund University Ecological Field Station (55°41′N, 13°26′E), where the experiments were performed. Garden warblers (*n*=23) were captured and brought into captivity between 31 August and 1 September 2024; robins (*n*=24) on 29 September 2024. Two separate experiments were performed in autumn 2024, one with garden warblers (16 days; 3–18 September), one with robins (17 days; 1–17 October), respectively. The individual birds were assigned to either the control group or the treatment group and to each house randomly.

Upon arrival, all birds were weighed to the nearest 0.1 g and the wing length was measured to the nearest 0.5 mm. Until the start of the experiments, the birds were kept indoors (local photoperiod) in individual cages, where they were given free access to water for drinking and bathing, and could feed *ad libitum*. We checked on the birds twice a day to ensure that they were all eating before we transferred them to the experimental cages. On the second day of the experiment, one garden warbler died overnight for unknown reasons and, therefore, the experiment went on with 22 individuals. The other 46 birds were all healthy throughout the experimental period and were released into the wild after the end of the experiment, all in good condition.

The experimental facility consisted of six wooden houses, each containing four cylindrical cages (550 mm diameter, 700 mm height), one for each bird, with non-transparent walls and a transparent net top (Ilieva et al., 2016). A circular perch in the centre of each cage was attached to a balance located right underneath the cage and two trays for placing food and water dispensers were also accessible from underneath the cage (Ilieva et al., 2016; Åkesson et al., 2021; Huffeldt et al., 2024). The temperature in the sheds was not regulated. While the weather was warm during the experiment with garden warblers (September; mean: +21°C, maximum: +35°C, minimum: +8°C), the temperature dropped during the experiment with robins (October; mean: +13°C, maximum: +21°C, minimum: +3°C).

The experiments were conducted in accordance with all relevant laws and regulations to work with animals. Birds were captured by a licensed ringer and permission for the experiments was given by the Malmö/Lund Ethical Committee for scientific research on animals (Dnr 5.8.18-12719/2017; 5.8.18-09591/2021; Sweden). The Swedish Board of Agriculture (Jordbruksverket) gave the authorisations for the experimental facility (Dnr 5.2.18-5398/16; 5.2.18-04121/2019) and the permit to work with animals (Dnr 5.2.18-10992/18). A permit to catch and ring birds (to Bo Petersson) was given from the Swedish Ringing Centre at the Natural History Museum in Stockholm and the Swedish Environmental Protection Agency.

### Photoperiod manipulation

The photoperiod manipulation was conducted following Åkesson et al. (2021) by turning the lights on and off via automated switches connected to timers following a set schedule. In each house, daylight was simulated using two LED lamps (colour temperature 4000 K, 100 W, 13,000 lm, Floodlight LED, Anslut) mounted on opposite walls and pointing towards the ceiling to mimic natural light. All houses were also provided with an IR lamp to simulate light at night, which was on during subjective night-time (colour temperature 1500 K, 75 W Night Heat Lamp, Exo-Terra).

In the three control houses (11 garden warblers, 12 robins), the natural photoperiod of that time of year at the experimental location (Lund, Sweden) was recreated; in the three houses with the experimental treatment (11 garden warblers, 12 robins), the photoperiod of Toulouse, southern France (43°60′N, 1°44′E) at that time of year was used (Ilieva et al., 2016; Ilieva et al., 2018). The range of photoperiod experienced by our garden warblers went from 13 h 38 min to 12 h 35 min in the control group and from 13 h 5 min to 12 h 24 min in the experimental group (difference in day length between the two groups went from 33 to 11 min, longer in the control group). The range of photoperiod experienced by robins went from 11 h 31 min to 10 h 23 min in the control group and from 11 h 42 min to 10 h 58 min in the experimental group (difference in day length between the two groups went from 11 to 35 min, longer in the experimental group). To set the schedule for the timers, we used https://www.timeanddate.com/ (last accessed: 10 October 2024).

### Data collection

The body mass and food intake of all birds were monitored daily throughout the experimental periods to gather fuelling data. The body mass was measured to the nearest 0.01 g with electronic balances (Precisa BJ410C; Precisa Gravimetrics AG, Dietikon, Switzerland) placed underneath each cage and connected to the perch (Ilieva et al., 2018). The measurements were always taken starting at 12:00 h (local time) in house 1 and proceeding to the last house (house 6), so that all measurements were taken 24 h apart for each individual. When the bird sat on the perch, its mass could be read on the balance without the observer being visible to the bird, making it possible to avoid any direct interaction between the animal and the person taking the measurements. During the daily visits, new food and water were provided for the birds, again by using the designated trays accessible from underneath the cages. Food consisted of exactly 20 g of live mealworms (*Tenebrio molitor*) provided daily for each bird, and the food intake was measured by weighing the leftover mealworms from the previous day at the time of rotation.

For the statistical analyses, the body mass measurements were used to calculate the daily scaled mass index (SMI) of each bird and thus its body condition (Langley et al., 2021). The SMI (Peig and Green, 2010) adjusts body mass accounting for body size, by standardising body mass at a fixed value of a linear body length measurement, in this case wing length, the structural trait most correlated with mass (Fransson et al., 2008), so it is a more correct way of assessing the body condition of a bird, compared with the raw mass data.

Activity data were collected by constantly filming the birds throughout the experiment with Axis P1427-LE network cameras fixed to the ceiling of each house, right above the cages and pointing down. The cameras recorded black and white video (6 frames s^−1^). After the experiment, a computer vision-generated method (Bianco et al., 2016, 2019a,b; Ilieva et al., 2018), was used to extract the activity data, defined as flying activity. The resulting activity data were expressed as the number of frames in which the bird was flying over 20 min intervals, reported as a ratio. The activity intervals were divided into day activity (lights on) and night activity (lights off) and the analyses focused on the latter.

### Data handling and statistical methods

All data handling, statistical analyses and plots were done with the software R (v.4.4.1, https://www.r-project.org/). The same analyses were conducted on both garden warblers and robins, to be able to compare them. Data handling and preprocessing were performed with the packages ‘dplyr’ (v.1.1.4, https://CRAN.R-project.org/package=dplyr), ‘lubridate’ (v.1.9.4, https://CRAN.R-project.org/package=lubridate) and ‘smatr’ (v.3.4.8, Warton et al., 2012) and included handling missing values using list-wise deletion (i.e. excluding rows with missing values). Before starting on any modelling, the data were visually explored with plots and histograms as a way of both carrying out quality control and detecting any trends.

The models chosen as the most apt to test differences in fuelling and activity between control and experimental groups were linear mixed-effects models (LMMs) and generalised linear mixed-effects models (GLMMs). These models are particularly well suited for the analysis of repeated measures and data with hierarchical structures, like the ones presented in this study. By incorporating random effects into the models, it was possible to account for individual differences between birds and the non-independence of data points. Furthermore, mixed-effects models allow for the inclusion of interactions between categorical and continuous variables and for the potential non-normality of the response variable.

The ‘lme4’ package (v.1.1-35.5, Bates et al., 2015) was used for the LMMs and the GLMMs. The models' assumptions were visually checked with the ‘performance’ package (v.0.12.4, Lüdecke et al., 2021) and with histograms of residuals. The models' summary statistics were accessed with base solutions and the ‘report’ package (v.0.5.9, https://CRAN.R-project.org/package=report) and ‘lmerTest’ package (v.3.1.3, Kuznetsova et al., 2017). The significance of the predictors was tested by comparing via a likelihood ratio test the LMMs with the relevant predictors and random effects to null-models, or intercept-only models, which included only the random factors while the predictors were fixed to 1 (Tredennick et al., 2021), thus allowing the initial hypotheses to be tested. The package ‘emmeans’ was used to calculate estimated marginal means of the models' predictions and 95% confidence intervals (CIs) for the plots (v.1.10.5, https://CRAN.R-project.org/package=emmeans), while the plots themselves were built with the package ‘ggplot2’ (v.3.5.1, https://CRAN.R-project.org/package=ggplot2).

#### Fuelling data

To test differences in body condition over time between the control and treatment group, we modelled the relationship between SMI (continuous) and an interaction between group (categorical) and the square of day (continuous). A random intercept for bird ID (categorical) accounted for repeated measures of each individual, while a random slope for day controlled for differences in initial SMI. The quadratic term for day accounted for the fact that there is a physiological limit to the body mass a bird can reach, creating a plateau. This model was selected based on likelihood ratio tests comparing it with the others we tried and because it addressed the study question best. After a similar process, to test differences in food intake over time between the two groups, we modelled the relationship between food intake (continuous) and an interaction between group and day, with bird ID as a random factor, i.e. random intercept. As the interaction term was non-significant in the robin model, it was later removed to avoid confusion in the estimates of the main factors (Engqvist, 2005).

For the garden warbler data, the food intake needed to be standardised with the scale() function by subtracting the mean from all values and then dividing them by the standard deviation. This was because this species, which had never been kept in the experimental facility before, turned out to be able to eat more than anticipated, with several individuals eating all the 20 g of mealworms on a few days, thus creating a truncated distribution of the food intake data.

#### Activity data

To test differences in the intensity of migratory activity between the control and experimental group over time, for the robin data, we modelled the relationship between mean time spent flying and an interaction between group and experimental night. For the garden warbler data, we chose a similar model, but we removed the interaction, as it was non-significant (Engqvist, 2005). Again, we controlled for repeated measures on the same individuals by including bird ID as a random intercept (Huffeldt et al., 2024). We investigated the mean activity over the 24 h, at daytime (lights on) and at night-time (lights off), but as both garden warblers and robins are nocturnal migrants (Fransson and Hall-Karlsson, 2008) and they show migratory restlessness at night, in this paper we only present the night activity models.

Moreover, many studies indicate that, when birds are held in captivity, the duration of their migratory activity is linked to the distance they would cover during their migration in the wild (Gwinner, 1986; Berthold, 1988). That is why we also modelled the duration of night activity, taking the artificial sunset as the starting time and considering the end as the time the birds reached the 90% threshold of their total night activity. To do this, as the dependent variable was non-Gaussian, we used a GLMM with a gamma distribution and the identity function as the link function, modelling the relationship between activity duration (continuous) and an interaction between group (categorical) and experimental night (continuous), with bird ID as the random factor once again. Again, we later removed the non-significant interaction from the garden warbler model.

There were no data points for food intake on the first day of the experiments, as for that it was necessary that the birds had been fed in the cages exactly 24 h before. Because of a problem with the timer schedule, on day 14 of the garden warbler experiment, sunset was delayed by some minutes, so activity data from that day were not included in the analyses. On day 11 of the robin experiment, there was a power-cut that lasted several hours, so both fuelling and activity data for that day had to be removed from the analyses.

## RESULTS

### Fuelling

#### Body condition

Overall, there was no significant difference in body condition between the control and the experimental group for either species (*P*=0.89 for garden warblers, Table 1; *P*=0.79 for robins, Table 2). There was, however, a significant difference in how body condition changed over time between groups in both experiments (*P*<0.05 for both garden warblers and robins, Tables 1 and 2, respectively). Considering the raw data, both groups of both species increased their body mass during the experiments. For robins, the trend was not pronounced enough to result in a significant increase in their SMI over time, while day square had a significant, although small, positive effect. As the effect of day was not significant, the effect of the quadratic term day square should be interpreted with caution. For garden warblers, the positive effect of day on SMI was substantial and highly significant (0.93±0.08, *P*<0.0001, Table 1).

**Table 1.** Output of the scaled mass index model for garden warblers

	Estimate	s.e.	d.f.	*t*-value	*P*-value	Group	Name	Variance	s.d.	Correlation
Fixed effects										
(Intercept)	18.58	0.75	24.21	24.72	**<0.0001**					
Exp. group	−0.15	1.06	24.27	−0.14	0.89					
Day	0.93	0.08	122.33	11.29	**<0.0001**					
Day^2^	−0.04	0.004	279.02	−9.94	**<0.0001**					
Exp. group:Day	−0.33	0.12	123.71	−2.80	**0.006**					
Exp. Day:Day^2^	0.02	0.005	279.16	3.16	**0.002**					
Random effects										
						Ring	(Intercept)	5.43	2.33	
							Day	0.25	0.16	−0.28
						Residual		0.60	0.77	

Statistically significant *P*-values (*P*<0.05) are in bold.

**Table 2.** Output of the scaled mass index model for European robins

	Estimate	s.e.	d.f.	*t*-value	*P*-value	Group	Name	Variance	s.d.	Correlation
Fixed effects										
(Intercept)	17.38	0.37	25.65	47.44	**<0.0001**					
Exp. group	0.14	0.52	25.62	0.27	0.79					
Day	−0.003	0.03	201.02	−0.08	0.93					
Day^2^	0.003	0.001	304.74	2.05	**0.04**					
Exp. group:Day	−0.09	0.05	199.21	−2.03	**0.04**					
Exp. Day:Day^2^	0.006	0.002	304.44	3.12	**0.002**					
Random effects										
						Ring	(Intercept)	1.45	1.20	
							Day	0.003	0.05	−0.29
						Residual		0.13	0.36	
										

Statistically significant *P*-values (*P*<0.05) are in bold.

**Fig. 1.**
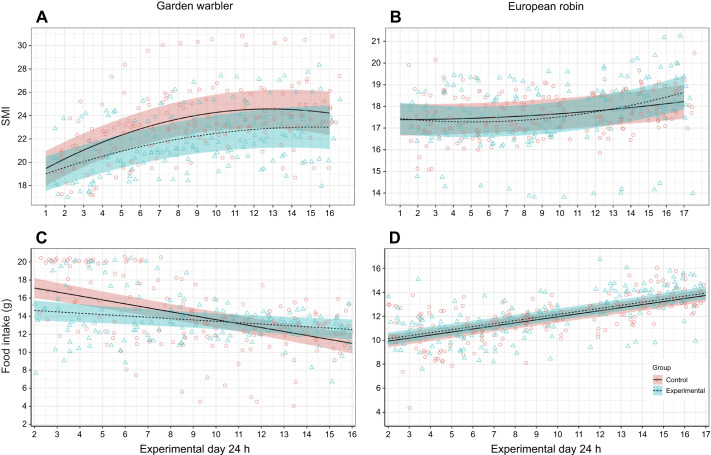
**Changes in scaled mass index (SMI) and food intake of garden warblers and robins in the photoperiod manipulation and control groups over time.** (A,B) SMI and (C,D) food intake for the control treatment group (garden warbler, *n*=11; European robin, *n*=12) and experimental treatment group (garden warbler, *n*=11; European robin, *n*=12). The pink circles (control) and blue triangles (experimental) represent the raw data points of each group. The black lines are the estimated marginal means for each group and the coloured areas are the 95% confidence intervals (CIs) of the estimated marginal means, built from the model predictions with the ‘emmeans’ package (Lenth, 2023).

For both species, the experimental group initially gained less mass than the control group, thus increasing their SMI less (Fig. 1A,B), as indicated by the negative interaction between experimental group and day in Tables 1 and 2. However, the interaction between experimental group and day square was positive, meaning that, in the long term, the experimental group could have maybe gained more mass than the control. For robins, the effect of the interaction between group and both day and day square was very small (−0.09±0.05 and 0.006±0.002, respectively), while for garden warblers, although small (−0.33±0.12 and 0.02±0.005), it was noticeable by just observing the plot.

At the end of the experiment, the control group of garden warblers on average had increased their SMI by 4.73 units (initial SMI=19.47, final SMI=24.20) and the experimental group by 4.00 units (initial SMI=19.01, final SMI=23.01). At the end of the experiment with robins, the control group had increased their SMI by only 0.84 units (initial SMI=17.38, final SMI=18.22) and the experimental group by 1.20 units (initial SMI=17.44, final SMI=18.64). The model explained over 90% of the variation for both species, but the variation explained by the fixed effects alone was 24% for garden warblers and 8% for robins, meaning most of the variation was due to differences between the individuals, which is the norm in this kind of analysis.

#### Food intake

In the garden warbler model for food intake, all estimates were highly significant (Table 3, Fig. 1C). For this species, the food intake decreased over time (−0.13±0.01 g, *P*<0.0001) and the birds in the experimental treatment group ate significantly less than the ones in the control treatment group (−0.91±0.26 g, *P*<0.001), although, over time, the decrease in food intake was greater for the control group (interaction between experimental group and day was 0.08±0.02 g, *P*<0.0001). The control group went from eating on average 17.1 g of mealworms on the first day to eating on average 11.2 g on the last day. The experimental group went from a food intake of 14.6 g to one of 12.7 g. The fixed effects alone explained 18% of the total variation in food intake data.

**Table 3.** Output of the standardised food intake model for garden warblers

	Estimate	s.e.	d.f.	*t*-value	*P*-value	Group	Name	Variance	s.d.
Fixed effects									
(Intercept)	1.24	0.18	88.77	6.77	**<0.0001**				
Exp. group	−0.91	0.26	90.35	−3.49	**0.0007**				
Day	−0.13	0.01	301.03	−8.61	**<0.0001**				
Exp. group:Day	0.08	0.02	301.21	3.97	**<0.0001**				
Random effects									
						Ring	(Intercept)	0.12	0.35
						Residual		0.70	0.84

Statistically significant *P*-values (*P*<0.05) are in bold.

The results were different for robins (Table 4, Fig. 1D), as no difference was found in food intake between the two treatment groups. The only significant effect was that of day, as both groups increased their food intake over time (0.25±0.01 g, *P*<0.0001). The control group went from eating 9.8 g of mealworms to 13.8 g on average, and the experimental group went from 10.2 g to 13.8 g, the same amount as the control group. The fixed effects alone explained 39% of the total variation in food intake data.

**Table 4.** Output of the food intake model for European robins

	Estimate	s.e.	d.f.	*t*-value	*P*-value	Group	Name	Variance	s.d.
Fixed effects									
(Intercept)	9.43	0.27	40.08	34.94	**<0.0001**				
Exp. group	0.20	0.33	21.83	0.60	0.55				
Day	0.25	0.01	328.99	16.87	**<0.0001**				
Random effects									
						Ring	(Intercept)	0.52	0.72
						Residual		1.78	1.33

Statistically significant *P*-values (*P*<0.05) are in bold.

### Activity

#### Overall activity

**Fig. 2.**
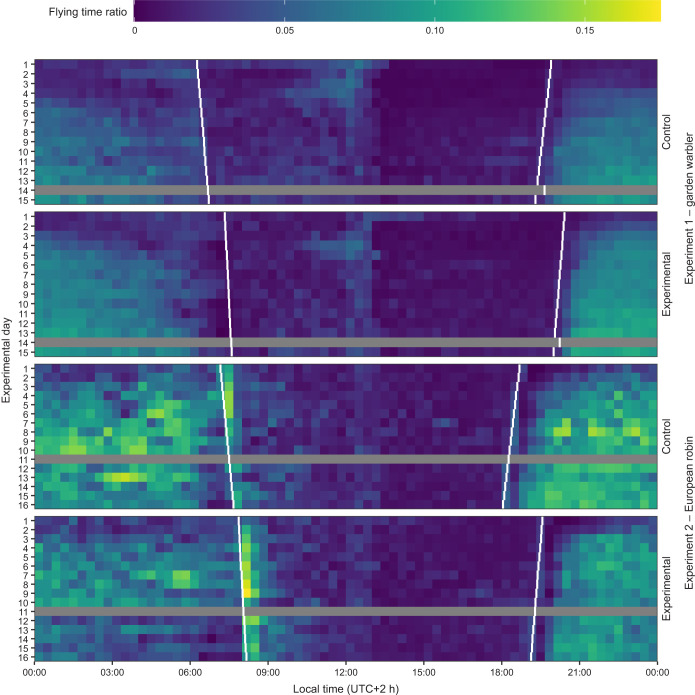
**Actograms showing the average activity throughout the day for garden warblers and European robins in the photoperiod manipulation and control groups.** Control treatment group: garden warbler, *n*=11; European robin, *n*=12; and experimental treatment group: garden warbler, *n*=11; European robin, *n*=12. The colour-coded horizontal cells illustrate the mean time spent flying over the 24 h period (from 00:00 h to 00:00 h the following day). The white lines indicate the artificial sunrise and sunset of each day. The grey cells indicate the days excluded from the analyses.

The actograms in Fig. 2 represent the average activity levels of all individuals of each treatment group over the 24 h throughout the experimental period. They are a good way of obtaining a general picture of the activity levels of the birds, although only qualitatively. Both species were mainly active at night, as expected from nocturnal migrants; thus, this activity can be interpreted as migratory restlessness. The afternoon, meanwhile, is the time of day when both species were the least active. Both species, but particularly garden warblers, only started showing migratory restlessness after the first few nights in captivity.

Robins reached higher activity peaks and not only were active at night but also expressed a substantial activity peak after sunrise. The increase in activity shown by garden warblers around noon coincided with our visits to feed and weigh the birds. The activity patterns of the two groups of garden warblers appeared similar, both increasing in intensity over time. For robins, conversely, the experimental treatment group decreased their activity over time, which did not seem to be the case for the control group, and showed overall lower activity levels.

#### Intensity of night activity

**Fig. 3.**
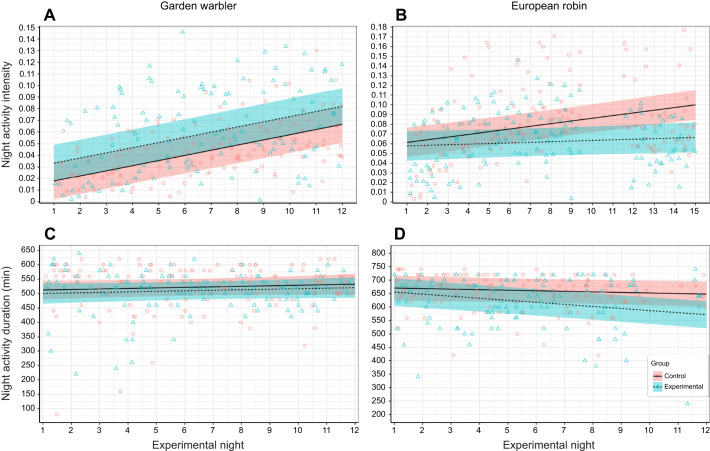
**Changes of mean night activity intensity and night activity duration of garden warblers and European robins in the photoperiod manipulation and control groups over time.** Night activity intensity (A,B) and duration (C,D) of the control treatment group (garden warbler, *n*=11; European robin, *n*=12) and experimental treatment group (garden warbler, *n*=11; European robin, *n*=12). The pink circles (control) and blue triangles (experimental) represent the raw data points of each group. The highest data points have been cut from the graphs to make them more easily readable. The black lines are the estimated marginal means for each group and the coloured areas are the 95% CIs of the estimated marginal means, built from the model predictions with the ‘emmeans’ package (Lenth, 2023).

The birds' mean night activity levels, measured as the individual mean time spent flying with lights off, was calculated on 12 nights for garden warblers and 13 nights for robins, because of the missing data for some of the experimental days, as mentioned in the Materials and Methods (Fig. 3A,B). For garden warblers, there was no significant difference in activity levels between the experimental treatment group and the controls, either as a whole or over time. The only significant effect was that of experimental night, which was positive for both groups (Table 5, upper half), meaning that the activity levels increased throughout the experiment. The activity levels of robins revealed significant differences between the two groups over time. The effect of experimental night on night activity was minimal, but significant and positive (2.75×10^−03^±4.41×10^−04^, *P*<0.0001, Table 6, upper half) and the night activity over time was lower in the experimental group compared with the control one (−2.12×10^−03^±6.23×10^−04^, *P*<0.001). The night activity model explained 52% of the variation, with the fixed effects covering 12% of it.

**Table 5.** Output of the night activity intensity and night activity duration models for garden warblers

	Estimate	s.e.	d.f.	*t*-value	*P*-value	Group	Name	Variance	s.d.
Intensity									
Fixed effects									
(Intercept)	1.35×10^−2^	7.65×10^−3^	2.25×10^1^	1.77	0.09				
Exp. group	1.54×10^−2^	1.05×10^−2^	2.00×10^1^	1.47	0.16				
Exp. night	4.42×10^−3^	2.83×10^−4^	2.41×10^2^	15.62	**<0.0001**				
Random effects									
						Ring	(Intercept)	0.0006	0.02
						Residual		0.0002	0.01
Duration									
Fixed effects									
(Intercept)	510.58	17.50		29.17	**<0.0001**				
Exp. group	−11.93	20.78		−0.57	0.57				
Exp. night	1.84	1.67		1.10	0.27				
Random effects									
						Ring	(Intercept)	525.67	22.93
						Residual		0.02	0.16

Statistically significant *P*-values (*P*<0.05) are in bold.

**Table 6.** Output of the night activity intensity and night activity duration models for European robins

	Estimate	s.e.	d.f.	*t*-value	*P*-value	Group	Name	Variance	s.d.
Intensity									
Fixed effects									
(Intercept)	5.87×10^−2^	7.62×10^−3^	3.37×10^1^	7.70	**<0.0001**				
Exp. group	−1.51×10^−3^	1.08×10^−2^	3.37×10^1^	−0.14	0.89				
Exp. night	2.75×10^−3^	4.41×10^−4^	2.86×10^2^	6.23	**<0.0001**				
Exp. group:Exp. night	−2.12×10^−3^	6.23×10^−4^	2.86×10^2^	−3.40	**0.0007**				
Random effects									
						Ring	(Intercept)	0.0005	0.02
						Residual		0.0006	0.02
Duration									
Fixed effects									
(Intercept)	672.75	24.08		27.93	**<0.0001**				
Exp. group	−9.55	33.56		−0.28	0.77				
Exp. night	−2.05	1.40		−1.46	0.14				
Exp. group:Exp. night	−5.59	1.88		−2.97	**0.003**				
Random effects									
						Ring	(Intercept)	1.55×10^03^	39.33
						Residual		1.61×10^−02^	0.13

Statistically significant *P*-values (*P*<0.05) are in bold.

#### Duration of night activity

The garden warblers' night activity duration, calculated taking the artificial sunset as the starting time and the time the birds reached the 90% threshold of their total night activity as the end, did not differ between the control and the experimental group, nor did it change significantly over time (Fig. 3C, Table 5, lower half). The model explained 100% of the variation, but the fixed effects explained only 15% of it. The variance was mostly explained by individual identity, and the within-individual variability was low, limiting the statistical power of the analysis, though the data suggest the absence of any significant treatment effect anyway. Results were different for robins (Fig. 3D), with the experimental treatment group reducing the duration of their night activity significantly more than the control group over time (−5.59±1.88 min, *P*<0.05, Table 6, lower half). The duration of night activity, just like the intensity, was calculated on 12 nights for garden warblers and on 13 nights for robins because of the missing data for some of the experimental days. This model explained 100% of the variation and the fixed effects covered up to 46% of it.

## DISCUSSION

We found the two study species responded to the photoperiod manipulation in different ways, generating hypotheses about strategy-dependent cue integration and species-specific adaptations to migration, although the differences in migration phase and temperature regime between the two experiments mean that a direct comparison was not possible. The robin, adapted to short-to-medium distance migration, wintering predominantly in the Mediterranean region, reacted by changing the nocturnal migratory activity in line with our predictions, while the fuelling did not change over the experimental period. The garden warbler, migrating to non-breeding areas in southern Africa, in contrast, did not change migratory activity as expected, but the fuelling was affected by the photoperiod, although not entirely in the way we predicted.

### European robins

#### Migratory activity in European robins

Our initial hypothesis on the activity patterns of robins was that the experimental group would reduce their migratory restlessness compared with the control group, if they perceived the virtual displacement to Southern France as reaching their wintering grounds (Pettersson and Lindholm, 1983; Fransson and Hall-Karlsson, 2008). The results we obtained confirmed our hypothesis. Both groups of robins significantly increased their night activity, i.e. migratory restlessness levels over time, albeit by a small degree. There were significant differences between the two groups over time, with the experimental group showing a smaller increase in their night activity. The robins also showed a peak of activity after sunrise, which is believed to be unrelated to migration (Åkesson et al., 2021). As birds that have exited from the migratory phenotype show it too, it could possibly be related to search activity for finding a feeding territory and defending it from other individuals, a well-known behaviour for both sexes in robins (Lack, 1939). The duration of the night activity presented a similar trend to the activity levels, such that the robins of the experimental group reduced the duration of their migratory activity significantly more than those of the control group over time. As noted, most of the variation in intensity of night activity was explained by inter-individual variability, with only 12% explained by fixed effects, while the percentage increased to 46% for the activity duration.

Given the small difference in terms of length of day and night between the two treatments, it is unlikely that this is the reason behind the results we got. Even though the robins of the experimental group experienced shorter nights, the extent of the difference was the same for the garden warblers, and it did not elicit the same results in that species. From these results, it appears that robins are sensitive to photoperiodic cues and react to them not only at the onset of their migration but also at the end, at least regarding the regulation of their migratory restlessness.

#### Fuelling in European robins

Regarding fuelling, our hypothesis for robins was that birds in the experimental group would fuel less than birds in the control group. This, however, was not confirmed by the results of either the SMI or the food intake data. There was no difference overall in SMI between the two treatment groups and, even though the interactions between experimental treatment and both day and day square were statistically significant, they were so small that they held no biological relevance. As for food intake, no difference was found between the two treatment groups, with the only significant effect being that of day, as both groups increased their food intake over time.

Considering only these results, it could appear that the robins were totally indifferent to the photoperiod manipulation, but this was disproved by the activity results. An alternative explanation, supported by previous observations (Gwinner and Czeschlik, 1978), is that, when birds are held in captivity, they might retain their migratory phenotype beyond the end of their migration in the wild, i.e. they tend to ‘overshoot’ (Helm and Liedvogel, 2024). It is likely that birds need a combination of factors to decide whether to exit from their migratory phenotype and that being caged, in an artificial setting, does not provide them with all the necessary cues informing them to stop (Gwinner and Czeschlik, 1978). These could include photoperiod, but also food availability, temperature, geomagnetic information, habitat and social cues (Fransson et al., 2001; Jenni and Schaub, 2003; Helm et al., 2009; Åkesson et al., 2017; Ilieva et al., 2018).

Furthermore, in the study by Åkesson et al. (2021) on the effect of photoperiod on the migratory phenotype of dunnocks and robins, the experimental robins, experiencing a day length extended by 2 h, did not change their fuelling compared with the control birds. This was not what the authors anticipated, as they expected that the birds would take advantage of the longer day length to forage more, as previously reported for other migratory bird species (Kvist and Lindström, 2000). Thus, in line with our results here, it appears that fuelling in robins is not controlled by photoperiod, but by other factors, such as the ones mentioned above. Hence, we suggest that control mechanisms for fuelling need to be investigated further, for robins as well as for other species.

Robins, migrating short distances, may be less time pressed when it comes to migration, compared with long-distance migrants, and therefore they can allow themselves to fuel more slowly and accumulate smaller fat reserves overall (Alerstam and Lindström, 1990; Engert et al., 2023). The feeding ecology of passerines at stopover sites plays an important role in their overall migration speed, as in many cases birds arriving at a stopover site must stay there several days before being able to accumulate fat (Rappole and Warner, 1976; Alerstam and Lindström, 1990). Pettersson (1983) observed that a robin needed about 10 days to regain the body mass it had when captured at a coastal stopover site, while other studies estimated that it took them around 6 days (Mehlum, 1983). Thus, it has been postulated that a good strategy for robins includes longer periods at stopover sites than they would need for a one-night flight, leaning towards a strategy of minimising the cost of transport rather than the time (Pettersson and Hasselquist, 1985; Dänhardt and Lindström, 2001). For robins, the perceived predation risk at night has also been shown to influence fuelling decisions, with a reduced fuelling rate to keep mass low when the predation risk was high (Bianco et al., 2025). Indeed, our robins in both treatment groups were gaining mass very slowly, despite steadily increasing their food intake, gaining around 1.5 g (8.8% increase in body mass) throughout the 2 week period in captivity. Thus, it is possible that they did not have the time to reach their optimal fuel load during the study period and, if the experiment ran longer, we could have possibly seen a different pattern towards the end, with the control group reaching a higher SMI than the experimental group.

### Garden warblers

#### Migratory activity in garden warblers

For garden warblers, we expected both treatment groups to be highly motivated to migrate, i.e. to retain high levels of migratory restlessness throughout the experimental period. Indeed, garden warblers of both groups expressed nocturnal Zugunruhe and the levels increased over time, without any significant difference between the two treatments. The duration of the migratory restlessness did not change over time, nor did it differ between groups.

Garden warblers of both treatment groups were almost exclusively active at night, except for a small activity peak at noon, most evident in the first experimental days and coinciding with the visits to the houses to feed and weigh the birds. It is probable that it was in fact caused by the visits themselves, but it is also possible that it was related to feeding or social behaviours (Helbig et al., 2010). The afternoon was the time when the birds were the least active. Garden warblers did not show migratory restlessness in the first few nights, probably because they were in a state of prioritising fuelling; thus, they were in ‘stopover mode’, which aligns with their fuelling curve. A similar effect of food intake and body condition on Zugunruhe has been reported for this species in previous work (Lupi et al., 2017). This was especially noticeable in our study, with the experimental group showing nocturnal Zugunruhe sooner than the control group. It could be that the experimental birds, because they interpreted the new photoperiod as either a spatial displacement to their stopover before the Mediterranean Sea crossing or as a temporal displacement later in the season felt more time pressed than the control birds (Lindström et al., 1994; Dänhardt and Lindström, 2001). As we did not expect any differences between the treatments, we cannot confirm that these results show that garden warblers were sensitive to the photoperiod manipulation and interpreted it in the way we predicted. Future research needs to address which cues are used by garden warblers to regulate their migratory activity during and at the end of migration.

#### Fuelling in garden warblers

We formulated two alternative hypotheses regarding fuelling in garden warblers: both treatment groups would be highly motivated to fuel, but either (1) they could have comparable fuelling rates or (2) the experimental group could fuel more efficiently than the controls. The reasoning was that the birds of the control group were at the beginning of their migration, while the birds of the experimental group had been virtually displaced, through photoperiod, to their stopover site before the crossing of ecological barriers such as the Mediterranean Sea and the Sahara Desert (Fransson and Hall-Karlsson, 2008). The experimental birds could have used this stopover to fuel substantially before these ecological barriers, but it was also possible that no difference would be observed between the two groups, given that various studies have found that the main fuelling for garden warblers occurs after the crossing of the Mediterranean Sea in coastal North Africa, right before the Sahara Desert (Bairlein, 1987; Fransson et al., 2006, 2008).

The results we obtained from the modelling of the SMI data pointed in the direction of the first hypothesis, as there was no significant difference in SMI between the two treatment groups overall, and the birds of the control group gained more mass on average. However, the significant interaction between experimental group and day square, reflected in the shape of the curve in Fig. 1, indicates that, had the experiment run longer, the alternative hypothesis could have possibly been confirmed, because the garden warblers of the experimental group could have surpassed the ones in the control group in terms of SMI, though this is just speculation and needs to be investigated further.

As expected from most long-distance migrants (Alerstam and Lindström, 1990), the garden warblers of both groups were very efficient in gaining mass in the first half of the experimental period (approximately 1 week) and then reached a plateau in body mass, as shown by the SMI model. This seems to have induced them to eat less in the second half of the experimental period and indeed the results of the food intake model show that the food intake decreased over time. The birds of the experimental treatment group ate significantly less than the ones in the control treatment, although, over time, the decrease in food intake was greater for the control treatment group.

The fact that 9 garden warblers of the control group and 2 of the experimental group ate all 20 g of mealworms at least once in the first few days caused a ceiling effect, as mentioned in the Materials and Methods, imbalanced towards the control group, which could confound the results of SMI and food intake. However, observing the trends of both models (Fig. 1A,C), it seems likely that, had the birds had more food available, these would be confirmed and possibly accentuated. Regarding the SMI model, it is possible that, without the ceiling effect, the control group would have still gained more mass, increasing the difference between the treatments. For the food intake, all terms were already significant and, without the ceiling effect, the difference between treatments both overall and over time would have probably been accentuated, not reversed. Unfortunately, another experiment with greater food rations would be necessary to assess this with certainty.

Despite eating less, the experimental treatment birds increased their SMI by a similar amount to the control treatment group, suggesting that a physiological change was occurring, though there is no direct proof. Many migratory bird species, particularly long-distance migrants, have been observed to undergo physiological changes as part of their migratory phenotype, to maximise the speed and efficiency of migration, some of which have the function of increasing the efficiency of food assimilation during the fuelling period and others of keeping body mass low during migratory flights (Piersma and Gill, 1998; Kvist and Lindström, 2003). This type of adaptation has also been found in garden warblers, with individuals increasing the daily amount of food they can metabolise during their fuelling period (Bairlein, 1987). Even though we did not find any difference in SMI between the two treatment groups overall, the significant difference in how SMI changed in the two groups over time could be an indication that the garden warblers were sensitive to the photoperiod manipulation, at least partially. Longer experiments in the future, possibly with a larger sample size, would help clarify this.

If garden warblers do incorporate photoperiod in their migratory programme during migration, it is probable that, to get a substantial increase in fuelling in the experimental group, the virtual displacement through photoperiod should be more southern, at the latitude of the north African coast. Many long-distance migratory songbirds breeding in Scandinavia have indeed been found to gather with non-random distributions in restricted, species-specific areas of the eastern Mediterranean, between Egypt and Turkey, assumed to be important stopover areas before the Sahara crossing in autumn (Fransson et al., 2005). Captures of first-year garden warblers stopping to fuel in Greece during their first autumn migration have shown that the body mass of the individuals arriving at the site varied a lot (13.3–32.8 g). This indicates that the birds reached that latitude in very diverse states of preparation regarding their fuelling status, though the average fuel load was <30% of their body mass (Fransson et al., 2006, 2008). The birds then stayed at the stopover site for 13–20 days and departed with an average fuel load of 100% of their body mass (Fransson et al., 2008). This suggests that the main fuelling stops in preparation for the crossing of the Sahara Desert occur very close to the desert and not before the crossing of the Mediterranean Sea. However, our experiment was designed to study the two species in the same set-up and anticipated photoperiodic distance (i.e. daylength change for the relevant period of year) and, therefore, both species were displaced to southern France, where the robins end migration.

Nevertheless, it is also possible that photoperiod is not a relevant cue for garden warblers during their migration. The migration of this species includes the crossing of the equator, and this passage happens around the equinox in autumn (Fransson and Hall-Karlsson, 2008), at a time when photoperiod does not change much between latitudes (Åkesson and Helm, 2020). Hence, at this time of year, photoperiod is not an ideal cue to extrapolate geographical latitudinal information. Garden warblers have been found to have an innate magnetic compass (Wiltschko and Gwinner, 1974), with the inherited ability to shift their orientation relative to the angle of inclination of the geomagnetic field when they cross the equator (Wiltschko and Wiltschko, 1992). Thus, it is reasonable to hypothesise that for this species, relying on geomagnetic cues above other factors may be adaptive; but *ad hoc* studies are needed to assess this. Still, it would be interesting to investigate whether garden warblers are more sensitive to photoperiod during their spring migration, as the long-distance movement to the breeding areas for this species mostly happens after the spring equinox (Fransson and Hall-Karlsson, 2008), and they show photoperiodic responsiveness in preparation for breeding (Gwinner et al., 1988).

Perhaps one of the most interesting aspects of the fuelling results we got is the exceptionally high fuelling rate that garden warblers exhibited. Among all the passerines that have been studied in the experimental facility used here (Ilieva et al., 2018, 2023; Bianco et al., 2019a,b; Åkesson et al., 2021, 2025; Engert et al., 2023; Huffeldt et al., 2024), garden warblers showed one of the highest fuelling rates starting right from the beginning of the experiment, gaining around 5 g (26.2% increase in body mass) in the first week, with some individuals eating all of the daily 20 g of mealworms in the first days. This seems to be an adaptive trait for a long-distance migrant with the aim to migrate as fast as possible, given that fuelling is the process that takes up the greatest time portion of migration (Hedenström and Alerstam, 1997), and it differentiates garden warblers from other warblers, such as the reed warbler (*Acrocephalus scirpaceus*) and the sedge warbler (*Acrocephalus schoenobaenus*) migrating shorter distances (Ilieva et al., 2023). Both *Acrocephalus* species winter in sub-Saharan Africa, but still, they show marked differences in their fuelling patterns (Ilieva et al., 2023). The ecological conditions under which songbirds have evolved to migrate can be expected to influence their fuelling rates (Engert et al., 2023). Given the notably high fuelling rate that garden warblers showed in this study, it is possible that this species always fuels at its maximum capacity throughout its migration and that they simply spend longer periods of time at stopover sites in which they need to fuel more extensively, i.e. before the crossing of ecological barriers (Fransson et al., 2008), instead of increasing their fuelling rate.

### Conclusions

Here, we have investigated the possible role of photoperiod input in the expression of the endogenous migratory programme of two passerine species during migration, something that, so far, has only been studied for a few species. Under the conditions tested, photoperiod integration during active migration appears species specific, but also limited in magnitude. The results we obtained highlight the complexity of the mechanisms controlling avian migration, in which differences between species of the same order and between distinct phases of migration within a species can be substantial (Åkesson and Helm, 2020; Ilieva et al., 2023). To build accurate predictions for how avian migrants will be impacted by climate change and shifting environments, it is essential to understand which cue is the most important for them in the different stages of migration and the degree of phenotypic plasticity and genetic variability linked to these traits.
